# Umbilical cord blood thyroid hormones are inversely related to telomere length and mitochondrial DNA copy number

**DOI:** 10.1038/s41598-024-53628-6

**Published:** 2024-02-07

**Authors:** Homa Ohadi, Parvin Khalili, Farzaneh Abasnezhad Kasrineh, Ozra Sadat Esmaeili, Faeze Esmaeili Ranjbar, Azita Manshoori, Mohammad Reza Hajizadeh, Zahra Jalali

**Affiliations:** 1 Department of Clinical Biochemistry, School of Medicine, Rafsanjan University of Medical Sciences, Rafsanjan, Iran; 2 Social Determinants of Health Research Centre, Rafsanjan University of Medical Sciences, Rafsanjan, Iran; 3 Department of Epidemiology, School of Public Health, Iran University of Medical Sciences, Tehran, Iran; 4 Molecular Medicine Research Center, Research Institute of Basic Medical Sciences, Rafsanjan University of Medical Sciences, Rafsanjan, Iran; 5 Department of Gynecology, School of Medicine, Rafsanjan University of Medical Sciences, Rafsanjan, Iran; 6 Non-Communicable Diseases Research Center, Rafsanjan University of Medical Sciences, Rafsanjan, Iran

**Keywords:** Biochemistry, Hormones

## Abstract

Hypothyroidism has been linked to reduced mortality rate and increased lifespan and health span. Telomere shortening, enhanced oxidative stress, and reduced cellular mitochondrial content are important hallmarks of aging shown to be related to age-associated diseases. It was proposed that the status of these markers in early life can be predictive of lifespan and the predisposition to certain age-associated disease in adulthood. Animal studies indicated that prenatal injection of thyroid hormones affects postnatal telomere length. Here, we sought to determine whether thyroid hormones TSH and fT4 are related to the telomere length, mitochondrial DNA copy number (mtDNAcn), and oxidative stress resistance marker GPX in the cord blood of newborns. In this study, we analyzed 70 mothers (18–42 years) and neonate dyads born in 2022 at the Nik Nafs maternity Hospital in Rafsanjan. The relative telomere length (RTL) and mtDNAcn were measured in the genomic DNA of cord blood leukocytes using real-time PCR. GPX enzyme activity was measured in the serum using colorimetric assays. In this study the correlation between these markers and the cord blood TSH and fT4 hormones were assessed using regression models. We found a reverse relationship between TSH levels and RTL in the cord blood of neonates. Additionally, our results displayed increased TSH levels associated with enhanced GPX activity. Regarding the mitochondrial DNA copy number, we found an indirect relationship between fT4 level and mtDNAcn only in male newborns. Future analyses of various oxidative stress markers, mitochondrial biogenesis status, telomerase activity, and the level of DNA damage are warranted to demonstrate the underlying mechanism of our observations.

## Introduction

Aging is a physiological process in which the power of cell growth and division decreases. The speed of aging is determined by genetic and environmental factors from the embryonic period to adulthood^1^. Telomere length, mitochondrial DNA copy number, and oxidative stress are important markers related to aging and aging-associated diseases such as hypertension, metabolic diseases such as insulin resistance and type 2 diabetes, as well as some cancers, and increased mortality^2–13^.

Research shows that the status of these markers at birth can be considered factors for longevity prediction and the predisposition to aging-related diseases in adulthood^14^. A recent prospective study determined that telomere length at the beginning of birth predicts telomere length in adulthood^14^. Many environmental factors, including smoking, drug use, antioxidant disorders, etc., can cause telomere shortening and telomerase activity disruption during a person’s life^15–17^. However, fewer studies are performed to find the factors that determine the length of newborn telomeres. These studies are limited to examining the effect of nutrition, psychological stress, and exposure to toxins during pregnancy^18,19^. Also, a few studies have investigated the effect of a mother’s condition during pregnancy on the number of mitochondria in the newborn^20,21^.

Thyroid disorders during pregnancy have transgenerational effects, which have been investigated only about the child’s neurodevelopment and cognitive and behavioral features^22–24^. Further studies are required to assess the effect of the prenatal level of thyroid hormones on other biological and health related characters.

Interesting results of several epidemiological studies and some animal studies have shown that hypothyroidism in adults and the elderly is associated with a decrease in mortality rate, an increase in lifespan and health span, and healthier aging^25–27^. These studies suggest a connection between thyroid hormones and aging hallmarks which requires further studies. An animal study titled “Born to be Young” has recently shown that prenatal injection of thyroid hormone in flycatchers causes telomere lengthening in early life^28^.Therefore, we conducted a study to investigate the relationship between the level of thyroid hormones TSH and fT4 in the cord blood of newborns with three hallmarks of aging, mtDNA content, telomere length, and the activity of antioxidant enzyme glutathione peroxidase as an indicator of oxidative stress.

## Material and methods

### Recruitment of participants, data collection, and ethics

In this study, we recruited 100 healthy pregnant women aged 18–42 who delivered newborns in 2022 at the Nik Nafs maternity Hospital in Rafsanjan. To obtain cord blood samples, mothers must be Iranian, complete a questionnaire interview and sign an informed consent form. Data used in the current study were collected from the health records of the maternity hospital for the mother and the newborn, as well as a detailed questionnaire used for an interview. The interview was performed by an obstetrician and a gynecologist. The data includes information on gestational thyroid diseases, blood pressure, and diabetes, maternal weight before pregnancy, maternal weight before delivery, maternal age, maternal opioid use, smoking**,** education, newborn anthropometry, newborn’s gender and gestational age at delivery, and the type of delivery). The study protocol was approved by the ethics committee of Rafsanjan University of Medical Sciences (Code of Ethics: IR.RUMS.REC.1400.223), and all the methods were performed following the relevant guidelines and rules of the ethics committee of Rafsanjan University of Medical Sciences. 70 mother-newborn pairs were included in our analysis after taking into account the following exclusion criteria. Mothers with BMI > 30, gestational diabetes, gestational thyroid diseases and hypertension, as well as mothers consuming cigarettes, alcohol, various drugs, and preterm newborns are excluded from the study.

### DNA extraction and telomere length measurements

We extracted about 100 μg of genomic DNA from the umbilical cord blood following the manufacturer’s instructions (Sina pure whole blood genomic DNA kit #EX6001, IRAN). Nanodrop spectrophotometer (DeNovix DS-11 Series, USA) was used to measure the quantity of DNA. Real-time polymerase chain reaction (PCR) was used to quantify the relative telomere length in DNA samples taken from umbilical cord blood leukocyte cells. This assay determines the ratio of the product of real-time PCR for the telomere (T) to a nuclear single copy gene (36B4d) (S) (T/S ratio) in a given sample. Then, we calculated RTL as T/S of the sample relative to the reference DNA used as a control sample in each PCR run. The PCR reaction was run in triplicates for each sample^29,30^. The Applied Biosystems step one plus Real-Time PCR machine and BIOSYSTEM Syber Green Mix qPCR reagent (#PB20.12.05, United Kingdom) were used. A template of 50 ng DNA utilized for each reaction.

The 5′ to 3′ sequences for the used primers were as follows: Telomere 1 (forward primer) TGTTAGGTATCCCTATCCCTATCCCTATCCCTATCCCTATCCCTAACA; Telomere 2 (reverse primer), ACACTAAGGTTTGGGTTTGGGTTTGGGTTTGGGTTAGTGT; 36B4d (forward primer); CAGCAAGTGGGAAGGTGTAATCC; 36B4d (reverse primer), CCCATTCTATCATCAACGGGTACCAA.

### mtDNA content analysis

mtDNA content in umbilical cord blood was determined by measuring the ratio of a mitochondrial gene copy number (MT-ND1) to a single copy genomic gene (36B4d) in each sample using the real-time polymerase chain reaction method^31,32^. For this experiment 100 ng DNA was used as a template for each reaction. 5′ to 3′ sequences for primers are as follows: ND-1 (forward primer) CCGACCTTAGCTCTCACCAT; ND-1(reverse primer), ATGCTCACCCTGATCAGAGG; 36B4d (forward primer); CAGCAAGTGGGAAGGTGTAATCC; 36B4d (reverse primer), CCCATTCTATCATCAACGGGTACCAA.

### Glutathione Peroxidase Activity Assessment

GPX enzyme activity was quantified in serum samples. First, whole blood was collected in clotted tubes, then it was centrifuged at 2500 rpm for 10 min at room temperature. After centrifugation, the resulting supernatant was collected as the serum which was stored at − 80 °C for the biochemical analyses. The kit (ZellBio # ZB-GPX-A96 Germany) was applied to quantify the antioxidant activity of glutathione peroxidase. The activity of this enzyme was quantified colorimetrically by ELISA Reader (BioTek, USA) at a wavelength of 412 nm.

### TSH and fT4 hormone measurement

This test is designed based on a sandwich ELISA, and the steps were carried out according to the kit (TSH measurement Ideal Diagnosing Atiyeh # 0524-96, Iran), (fT4 measurement Ideal Diagnosing Atiyeh #3124-96 Iran).

### Statistical analysis

Quantitative variables were described as the mean ± standard deviation or median [IQR] as appropriate, and the categorical variables as the frequency and percentage. The characteristics of individuals were compared across the groups of telomere length, mitochondrial DNA copy number, and GPX antioxidant enzyme activity, using the chi-square or Fisher’s exact test for the categorical variables and independent t-test for normally distributed quantitative variables and the Mann–Whitney U test for not normally distributed quantitative variables. In addition, we used crude and adjusted dichotomous logistics regression analysis to estimate odds ratios (ORs) with 95% confidence intervals (CIs) for the associations between relative telomere length, mitochondrial DNA copy number, and GPX antioxidant enzyme activity level with thyroid hormone levels. Confounder variables such as mother’s age, mother’s weight before pregnancy, baby’s weight, gestational age, baby’s gender, and mother’s education were identified using relevant epidemiological texts and based on subject matter knowledge. Next, separate models at the bivariate level were run to obtain variables related to telomere length, mitochondrial DNA copy number, and GPX enzyme activity. After that, variables with a value of *p* < 0.25. Were considered for the analysis and included in the multivariable model. All analyses were performed through State V.14. All *p*-values are two-sided, and *p*-values < 0.05 and 95% confidence intervals not including 1 were considered statistically significant.

### Ethics approval and consent to participate

This study was approved by the Ethics Committee of Rafsanjan University of Medical Sciences (Code of Ethics: IR.RUMS.REC.1400.223). All procedures of this research were conducted under the supervision of the Ethics Committee of Rafsanjan University of Medical Sciences. Signed consent was obtained from all participant mothers.

## Results

In the current study, relative telomere length, mitochondrial DNA copy number, and GPX enzyme activity in the umbilical cord blood of the neonates were measured, and their relationship with the level of thyroid hormones TSH and fT4 were evaluated.

### Descriptive analyses

Neonates were divided into two groups based on the median of GPX enzyme activity (250.04), which are referred to as the group with lower enzyme activity than the median (GPX_low), and the group with equal or higher enzyme activity than the median (GPX_high). As shown in Table 1, a significant difference was observed in serum TSH levels between the two GPX enzyme activity groups (*p*-value = 0.037). No significant difference was found between the two GPX enzyme activity groups in maternal age (*p*-value = 0.43), neonatal weight (*p*-value = 0.76), pre-pregnancy maternal weight (*p*-value = 0.92), gestational age at delivery (*p*-value = 0.93), and fT4 hormone (*p*-value = 0.09). Additionally, the relationship between qualitative variables such as the mother’s education (below diploma/diploma and above), birth order of the neonate (first-born/second-born/third-born and above), neonate gender(male/female), infertility history(yes/no), high fat diet (yes/no), and stress during pregnancy (financial problems/job related problems/family disputes/death of loves ones) with GPX enzyme activity were assessed. The results showed no significant association between the mother’s education (*p*-value = 0.35), the birth order of the neonate (*p*-value = 0.58), neonate gender (*p*-value = 0.31), infertility history (*p*-value = 1.0), high fat diet (*p*-value = 1.0), and stress during pregnancy (*p*-value = 0.89) with GPX enzyme activity.

**Table 1:** Baseline characteristics of the study population categorized by the level of GPX enzyme activity.

Variable	Glutathione peroxidase-Low	Glutathione peroxidase –High	*p*-value
Thyroid stimulating hormone (µIU/ml) Median (IQR)	6 (4.2–7)	6.4 (5.2–10)	**0.037**^**a**^
Mother age(years) Mean ± SD	32.39 ± 5.34	31.39 ± 4.93	0.43^c^
Neonate weight(g) Mean ± SD	3122.12 ± 329.96	3148.79 ± 391.64	0.76^c^
Pre-pregnancy maternal weight) kg) Mean ± SD	64.70 ± 9.41	64.94 ± 9.81	0.92^c^
Gestational age (weeks) Mean ± SD	38.61 ± 0.80	38.63 ± 0.89	0.93^c^
Free T4 hormone (pmol/l) Mean ± SD	7.85 ± 3.18	9.15 ± 3.14	0.099^c^
Mother’s education No (%)			
Low (below diploma)	7(21.21)	3(9.09)	0.35^b^
Middle (high diploma and postgraduate diploma)	17(51.52)	18(54.55)	
High (Bachelor’s degree and higher)	9(27.27)	12(36.36)	
Birth order of the neonate No (%)			
First born	9(27.27)	8(24.24)	0.58^d^
Second born	14(42.42)	18(54.55)	
Third born and above	10(30.30)	7(21.21)	
Neonate gender No (%)			
Male	21(63.64)	17(51.52)	0.31^d^
Female	12(36.36)	16(48.48)	
Infertility history No (%)			
Yes	3(9.09)	2(6.06)	1.0^b^
No	30(90.91)	31(93.94)	
High fat diet No (%)			
Yes	1(3.03)	1(3.03)	1.0^b^
No	32(96.97)	32(96.97)	
Stress during pregnancy No (%)			
Financial problems	25(75.76)	23(69.70)	0.89^b^
Job related problems	3(9.09)	3(9.09)	
Family disputes	1(3.03)	2(6.06)	
Death of loved ones	2(6.06)	4(12.12)	

High-fat diet was defined as self-reported consumption of high fat food every day.

*SD* Standard deviation.

*IQR* The interquartile range.

^a^Wilcoxon rank-sum (Mann–Whitney) test.

^b^Fisher exact test/χ^2^ test.

^c^t-test.

^d^Chi^2^ test.

Significant values are in bold.

Neonates were divided into two groups based on the median relative telomere length (1.79), which are referred to as the group with lower relative telomere length than the median (Telomere_low) and the group with equal or higher relative telomere length than the median (Telomere_high). Since the median relative telomere length in mothers who had natural childbirth had a significant difference with mothers who had a cesarean section, and the majority of our population had undergone cesarean section (80%), the analysis was only conducted on mothers who had a cesarean section to remove the effect of delivery type on our results. As shown in Table 2, no significant association was found between relative telomere length and any of the quantitative variables including fT4 hormone level (*p*-value = 1.0), TSH hormone level (*p*-value = 0.34), maternal age (*p*-value = 0.44), neonatal weight (*p*-value = 0.76), pre-pregnancy maternal weight (*p*-value = 0.76), and gestational age at delivery (*p*-value = 0.86). Additionally, no significant association was found between any of the qualitative variables including neonate gender (*p*-value = 0.1), mother’s education (below diploma/diploma and above) (*p*-value = 0.34), birth order of the neonate (first-born/second-born/third-born and above) (*p*-value = 0.68), infertility history(yes/no) (*p*-value = 1.0), high fat diet(yes/no) (*p*-value = 0.50), and stress during pregnancy(financial problems/job related problems/family disputes/death of loves ones) (*p*-value = 0.65) with the relative telomere length.

**Table 2:** Baseline characteristics of the study population categorized by the relative telomere length.

Variable	Relative telomere length-Low	Relative telomere length -High	*p*-value
Thyroid stimulating hormone (µIU/ml) Median (IQR)	6.1 (4.8–9)	5.7 (5–6.5)	0.29^a^
Mother age (years) Mean ± SD	32.48 ± 4.46	31.41 ± 5.93	0.44
Neonate weight (g) Mean ± SD	3127.24 ± 354.67	3097.59 ± 393.18	0.76
Pre- pregnancy maternal weight (kg) Mean ± SD	65.38 ± 9.08	66.14 ± 10.03	0.76
Gestational age (weeks) Mean ± SD	38.47 ± .51	38.51 ± .90	0.86
Free T4 hormone (pmol/l) Mean ± SD	8.41 ± 3.49	8.41 ± 3.51	1.0
Mother’s education No (%)			
Low (below diploma)	4 (13.79)	2 (6.90)	0.34^b^
Middle (high diploma and postgraduate diploma)	18 (62.07)	15 (51.92)	
High (Bachelor’s degree and higher)	7 (24.14)	12 (41.38)	
Birth order of the neonate No (%)			
First born	8 (27.59)	7 (24.14)	0.68^b^
Second born	15 (51.72)	13 (44.83)	
Third born and above	6 (20.69)	9 (31.03)	
Neonate gender No (%)			
Male	14 (48.28)	20 (68.97)	0.1^b^
Female	15 (51.72)	9 (31.03)	
Infertility history No (%)			
Yes	2 (6.90)	3 (10.34)	1.0^b^
No	27 (93.10)	26 (89.66)	
High fat diet No (%)			
Yes	2 (6.90)	0 (00.00)	0.50^b^
No	27 (93.10)	29 (100)	
Stress during pregnancy No (%)			
Financial problems	18 (62.07)	23 (79.31)	0.65^b^
Job related problems	3 (10.34)	1 (3.45)	
Family disputes	2 (6.90)	2 (6.90)	
Death of loved ones	3 (10.34)	2 (6.90)	

High-fat diet was defined as self-reported consumption of high fat food every day.

*SD* Standard deviation.

*IQR* The interquartile range.

^a^Wilcoxon rank-sum (Mann–Whitney) test.

^b^Fisher Exact Test/χ^2^ test.

^c^t-test.

Neonates were divided into two groups based on the median mtDNA copy number (3.98), which are referred to as the group with a lower mtDNA copy number than the median (mtDNA_low) and the group with equal or higher mtDNA copy number than the median (mtDNA_high). As depicted in Table 3, no significant association was found between mtDNA copy number and any of the quantitative variables including maternal age (*p*-value = 0.27), neonatal weight (*p*-value = 0.19), fT4 hormone level (*p*-value = 0.39), TSH hormone level (*p*-value = 0.62), pre-pregnancy maternal weight (*p*-value = 0.70), and gestational age at delivery (*p*-value = 0.99). Additionally, no significant association was found between any of the qualitative variables including the birth order of the neonate (first-born/second-born/third-born and above) (*p*-value = 0.15), mother’s education (below diploma/diploma and above) (*p*-value = 0.67), infertility history(yes/no) (*p*-value = 1.0), high fat diet(yes/no) (*p*-value = 0.49), and stress during pregnancy(financial problems/job related problems/family disputes/death of loves ones) (*p*-value = 0.49) with mtDNA copy number between the two groups. However, a significant association was observed between neonate female gender and mtDNA copy number (*p*-value = 0.008). In individuals with mtDNA_low, 74.29% of the neonates were male and 25.71% were female, while in individuals with mtDNA_high, 42.86% of the neonates were male and 57.14% were female.

**Table 3:** Baseline characteristics of the study population categorized by the mitochondrial DNA copy number.

Variable	Mitochondrial DNA copy number—low	Mitochondrial DNA Copy number—high	*p*-value
Thyroid stimulating hormone (µIU/ml) Median (IQR)	6.1(4.6–8.5)	6.2(5–9)	0.62^a^
Mother age (years) Mean ± SD	32.54 ± 4.88	31.17 ± 5.44	0.27^b^
Neonate weight(g) Mean ± SD	3178.43 ± 329.30	3075.14 ± 371.19	0.19^c^
Pre- pregnancy maternal weight(kg) Mean ± SD	64.41 ± 9.93	65.31 ± 9.35	0.70^c^
Gestational age(weeks) Mean ± SD	38.59 ± .77	38.58 ± .90	0.99^c^
FreeT4 hormone(pmol/l) Mean ± SD	8.71 ± 3.51	8.03 ± 3.03	0.39^c^
Mother’s education No (%)			
Low (below diploma)	4 (11.43)	6 (17.14)	0.67^b^
Middle (high diploma and postgraduate diploma)	21 (60)	17 (48.57)	
High (Bachelor's degree and higher)	10 (28.57)	12 (34.29)	
Birth order of the neonate No (%)			
First born	7 (20)	11 (31.43)	0.15^b^
Second born	15 (42.86)	18 (51.43)	
Third born and above	13 (37.14)	6 (17.14)	
Neonate gender No (%)			
Male	26 (74.29)	15 (42.86)	**0.008**^**b**^
Female	9 (25.71)	20 (57.14)	
Infertility history No (%)			
Yes	2 (5.71)	3 (8.57)	1.0^b^
No	33 (94.29)	32 (91.43)	
High fat diet No (%)			
Yes	2 (5.71)	0 (0)	0.49^b^
No	33 (94.29)	35 (100)	
Stress during pregnancy No (%)			
inancial problems	33 (65.71)	27 (77.14)	0.49^b^
Job related problems	4 (11.43)	2 (5.71)	
Family disputes	3 (8.57)	1 (2.86)	
Death of loved ones	4 (11.43)	2 (5.71)	

high-fat diet was defined as self-reported consumption of high fat food every day.

*SD* Standard deviation.

*IQR* The interquartile range.

^a^ Wilcoxon rank-sum (Mann–Whitney) test.

^b^ Fisher Exact Test /χ^2^ test.

^c^ t-test.

Significant values are in bold.

### Logistic regression analyses

The relationship between aging parameters and the level of TSH and fT4 hormones in the blood was assessed using unadjusted and adjusted logistic regression analysis. In the adjusted model 1, the effects of neonate gender, mother’s education, and neonate weight were adjusted, and in model 2, in addition to the variables in model 1, gestational age and mother’s weight before pregnancy were also adjusted. Table 4 shows the results of single and multiple-variable logistic regression models for investigating the relationship between the level of antioxidant enzyme GPX activity and thyroid hormones (TSH-fT4). Regarding the fT4 hormone, the unadjusted model did not show a significant relationship between this hormone and GPX enzyme activity (OR: 1.14, 95% CI = 0.97–1.31, *p*-value = 0.10). Also, in models 1 and 2, a significant relationship was not observed according to Table 4 (model 1 OR = 1.16, 95% CI = 0.98–1.37, *p*-value = 0.07) (model2 OR = 1.17, 95% CI = 0.98–1.39, *p*-value = 0.08). However, a significant association was observed for TSH hormone in all three logistic regression models. According to the unadjusted model, the odds of having a higher TSH was 20% higher in individuals with GPX_high compared to those with GPX_low. In other words, the GPX_high group has 20% higher odds of having a higher TSH (OR = 1.20, 95% CI = 1.02–1.42, *p*-value = 0.025). In the adjusted model 1, it was shown that with an increase in TSH level in individuals, the odds of having GPX_high compared to GPX_low is 22% higher (OR = 1.22, 95% CI = 1.03–1.45, *p*-value = 0.021), and in the adjusted model 2, the odds of having a higher TSH in the GPX_high group compared to the GPX_low group is 26% higher (OR = 1.26, 95% CI = 1.04–1.53, *p*-value = 0.019) (Table 4).

**Table 4:** Estimated unadjusted and adjusted odds ratios for thyroid hormones, as predicted by GPX antioxidant enzyme activity.

GPX_high VS. GPX_low	Unadjusted model		Adjusted model 1		Adjusted model 2	
	OR (95% CI)	*p*-value	OR (95% CI)	*p*-value	OR (95% CI)	*p*-value
Level of thyroid stimulating hormone (µIU/ml)	1.20 (1.02–1.42)	**0.02**	1.22 (1.03–1.45)	**0.02**	1.26 (1.04–1.53)	**0.01**
Level of free T4 hormone (pmol/l)	1.14 (0.97–1.34)	0.10	1.16 (0.98–1.37)	**0.07**	1.17 (0.98–1.39)	**0.08**

*CI* Confidence interval.

*Model 1* Adjusted for neonate gender, mother’s education and neonate weight.

*Model 2* Adjusted for neonate gender, mother’s education, neonate weight, gestational age and pre-pregnancy maternal weight.

Significant values are in bold.

Table 5 shows the results of the logistic regression models examining the relationship between relative telomere length and thyroid hormone levels (TSH-fT4). In the unadjusted model, no significant relationship was observed between relative telomere length and thyroid hormone levels. However, in adjusted model 1, which was adjusted for neonate gender, neonate weight, and mother’s education, a significant relationship between TSH levels and relative telomere length was observed (OR = 0.78, 95% CI = 0.98–0.62, *p*-value = 0.03). Additionally, in adjusted model 2, which was adjusted for the variables in model 1 as well as gestational age at pregnancy and maternal weight before pregnancy, a significant relationship was also observed between TSH levels and relative telomere length. The relationship was such that the chance of having a higher TSH level in the Telomere_low group compared to the Telomere_high group increased by 63% (OR = 0.37, 95% CI = 0.96–0.55, *p*-value = 0.02) (Table 5). Regarding thyroid hormone fT4 and the relative telomere length, no significant relationship was observed in the unadjusted model (OR = 1.00, 95% CI = 0.86–1.16, *p*-value = 1.00), adjusted model 1 (OR = 1.00, 95% CI = 0.86–1.18, *p*-value = 0.96), or adjusted model 2 (OR = 1.00, 95% CI = 0.84–1.18, *p*-value = 0.98).

**Table 5:** Estimated unadjusted and adjusted odds ratios for thyroid hormones, as predicted by relative telomere length.

RTL_high VS. RTL low	Unadjusted model		Adjusted model 1		Adjusted model 2	
	OR (95% CI)	*p*-value	OR (95% CI)	*p*-value	OR (95% CI)	*p*-value
Level of thyroid stimulating hormone (µIU/ml)	0.84(0.70–1.02)	0.08	0.78(0.62–0.98)	**0.03**	0.37(0.55–0.96)	**0.02**
Level of free T4 hormone (pmol/l)	1.00(0.86–1.16)	1.00	1.00(0.86–1.18)	0.96	1.00(0.84–1.18)	0.98

*CI* Confidence interval.

*Model 1* Adjusted for neonate gender, mother’s education and neonate weight.

*Model 2* Adjusted for neonate gender, mother’s education, neonate weight, gestational age and pre-pregnancy maternal weight.

Significant values are in bold.

Table 6 shows the results of logistic regression models, investigating the relationship between the number of mitochondrial DNA copies and thyroid hormone levels (TSH-fT4). As the results of the unadjusted model indicate, there is no significant statistical relationship between the number of mitochondrial DNA copies and TSH hormone levels (OR = 1.04, 95% CI = 0.91–1.18, *p*-value = 0.59), as well as fT4 hormone levels (OR = 0.94, 95% CI = 0.80–1.08, *p*-value = 0.38). Moreover, after adjusting for variables neonate gender, mother’s education, and neonate weight using the multivariable model 1 (adjusted model 1), no significant relationship was observed between the number of mitochondrial DNA copies and TSH hormone levels (OR = 1.03, 95% CI = 0.90–1.18, *p*-value = 0.63) or fT4 hormone levels (OR = 0.90, 95% CI = 0.77–1.06, *p*-value = 0.32). Furthermore, in the adjusted model 2, no significant relationship was observed between the number of mitochondrial DNA copies and TSH hormone levels (OR = 1.04, 95% CI = 0.90–1.20, *p*-value = 0.60) or fT4 hormone levels (OR = 0.92, 95% CI = 0.78–1.08, *p*-value = 0.29).

**Table 6:** Estimated unadjusted and adjusted odds ratios for thyroid hormones, as predicted by relative mitochondrial DNA copy number.

mtDNA_high VS. mtDNA_low	Unadjusted model		Adjusted model 1		Adjusted model 2	
	OR (95% CI)	*p*-value	OR (95% CI)	*p*-value	OR (95% CI)	*p*-value
Level of thyroid stimulating hormone (µIU/ml)	1.04 (0.91–1.18)	0.59	1.03 (0.90–1.18)	0.63	1.04 (0.90–1.20)	0.60
Level of free T4 hormone (pmol/l)	0.94 (0.80–1.08)	0.38	0.90 (0.77–1.06)	0.23	0.92 (0.78–1.08)	0.29

*CI* Confidence interval.

*Model 1* Adjusted for neonate's gender, mother’s education and neonate's weight.

*Model 2* Adjusted for neonate's gender, mother’s education, neonate's weight, gestational age and pre-pregnancy maternal weight.

Given that sex was an important and influential factor in the number of mtDNA copies, we conducted a sensitivity analysis to control for the residual effect of sex. According to Table 7 and the results of the unadjusted model, there was no significant association between the level of TSH hormone and the number of mtDNA copies in either girl (OR = 1.01, 95% CI = 0.82–1.24, *p*-value = 0.96) or boys (OR = 1.06, 95% CI = 0.90–1.25, *p*-value = 0.46). In adjusted model 1, which was adjusted for the mother’s education and neonate weight, there was no significant association between the level of TSH hormone and the number of mtDNA copies in either boy (OR = 1.05, 95% CI = 0.89–1.24, *p*-value = 0.54) or girls (OR = 1.01, 95% CI = 0.81–1.26, *p*-value = 0.94). In adjusted model 2, which was adjusted for gestational age at pregnancy and pre-pregnancy weight, as well as the variables in adjusted model 1, there was still no significant association between the level of TSH hormone and the number of mtDNA copies in either boy (OR = 1.06, 95% CI = 0.87–1.29, *p*-value = 0.55) or girls (OR = 1.01, 95% CI = 0.80–1.27, *p*-value = 0.96). Regarding the fT4 hormone, in girls, in the unadjusted model (OR = 1.05, 95% CI = 0.84–1.31, *p*-value = 0.67), adjusted model 1 (OR = 0.92, 95% CI = 0.70–1.21, *p*-value = 0.54), and adjusted model 2 (OR = 0.93, 95% CI = 0.70–1.22, *p*-value = 0.59), there was no significant association between the level of fT4 hormone and the number of mtDNA copies. However, in boys, the results of the unadjusted model showed that with an increase in the level of fT4 hormone, the chance of having a higher number of mtDNA copies compared to individuals with a lower number of mtDNA copies (mtDNA-low) decreased by 23% (OR = 0.77, 95% CI = 0.61–0.98, *p*-value = 0.034). In the adjusted model 1, after adjusting for neonate weight and mother’s education, there was a significant association with an increase in the level of fT4 hormone, the chance of having a higher number of mtDNA copies in boys compared to individuals with a lower number of mtDNA copies decreased by 22% (OR = 0.78, 95% CI = 0.61–0.99, *p*-value = 0.038). Additionally, in adjusted model 2, after adjusting for the variables in adjusted model 1 as well as maternal pre-pregnancy weight and age at pregnancy, there was still a significant association with an increase in the level of fT4 hormone, the chance of having a higher number of mtDNA copies in boys compared to individuals with a lower number of mtDNA copies decreased by 22% (OR = 0.77, 95% CI = 0.60–0.99, *p*-value = 0.04) (Table 7).

**Table 7:** Estimated unadjusted and adjusted odds ratios for thyroid hormones, as predicted by the relative mitochondrial DNA copy number stratified by neonate gender.

mtDNA_high	Unadjusted model		Adjusted model 1		Adjusted model 2	
	OR (95% CI)	*p*-value	OR (95% CI)	*p*-value	OR (95% CI)	*p*-value
Level of thyroid stimulating hormone (µIU/ml)						
Girl	1.01 (0.82–1.24)	0.96	1.01 (0.81–1.26)	0.94	1.01 (0.80–1.27)	0.96
Boy	1.06 (0.90–1.25)	0.46	1.05 (0.89–1.24)	0.54	1.06 (0.78–1.29)	0.55
Level of free T4 hormone (pmol/l)						
Girl	1.05 (0.84–1.31)	0.67	0.92 (0.70–1.21)	0.54	0.93 (0.70–1.22)	0.59
Boy	0.77 (0.61–0.98)	**0.03**	0.78 (0.61–0.99)	**0.03**	0.77 (0.60–0.99)	**0.04**

*CI* Confidence interval.

*Model 1* Adjusted for mother’s education and neonate weight.

*Model 2* Adjusted for mother’s education, neonate weight, gestational age and pre-pregnancy maternal weight.

Significant values are in bold.

Finally, we also examined the correlation between mtDNAcn and RTL using Spearman’s correlation test. The results of this test showed that the correlation between mtDNAcn and RTL is not statistically significant (*p* = 0.415).

## Discussion

Previous epidemiological studies have indicated hypothyroidism in the elderly to be associated with reduced mortality rate, and increased lifespan and health span^25–27^. It was explained by increased respiration rate and cellular metabolic activities induced by thyroid hormones, leading to higher oxidative stress and increased mitochondrial DNA damage. Telomere shortening and a decline in mtDNA copy number in cells are two important hallmarks of aging and age-associated diseases^11–13^. It has been shown that the status of these markers early in life can be used to predict lifespan and the predisposition to certain age-associated diseases in the future^14^. Here we sought to determine whether thyroid hormones TSH and fT4 are related to the relative telomere length, mtDNA copy number, and oxidative stress resistance marker GPX in the cord blood of neonates.

In the present study, we found a reverse relationship between TSH levels and the relative telomere length in the cord blood of neonates. In this regard, only two animal studies with opposing results have been performed previously by the same research group. They found that supplementation of thyroid hormones during the pre-natal period leads to shorter telomere length during the juvenile age of tits^33^, but in contrast to flycharts, it results in longer postnatal telomeres. They did not observe the telomere shortening in association with a significant difference in the mitochondrial density or oxidative stress resistance biomarkers. They suggested the increased growth induced by thyroid hormones as the potential mediator factor for shorter telomeres in TH-supplemented tits^28^. Concerning their opposite results in tits and flycharts, they suggested that the cross-species differences might be the explanation. Here, we found in human neonates that higher thyroid hormone TSH in the cord blood is associated with shorter telomere length. In parallel, we found increased TSH levels to be associated with increased GPX activity in the serum of cord blood. Future investigation is required to assess whether what we observed may be due to increased oxidative stress caused by higher TSH hormone, adversely affecting the telomere length and triggering a secondary protective response by increasing antioxidant activity in the cord blood. Therefore, experiments are warranted to assess this hypothesis by measuring multiple oxidative stress and antioxidant capacity markers of cord blood serum, and DNA damage indicators of cord blood cells.

Regarding the mitochondrial DNA copy number, we found that only in male newborns, higher fT4 level is significantly associated with lower mitochondrial DNA copy numbers in the cord blood cells. In a previous study, it was reported that higher fT4 levels in cord blood are associated with higher mitochondrial DNA copy numbers in the placenta of neonates which was explained by enhanced mitochondrial biogenesis regulated by thyroid hormones^34^. Further investigation is required to assess whether the difference between cord blood cells and the placenta may be the reason for the difference in our results. Research has shown that environmental factors such as exposure to pollution, tobacco smoking and drug consumption^35^ during pregnancy impacts the telomere length and the mitochondrial DNA copy number at birth^36–40^.

In this study, we excluded smoking and drug consuming mothers to remove ethe impact of these environmental factors from our analysis. We also collected data on factors such as high fat diet and high sugar diet and type of stress during pregnancy which did not show a significant association with the telomere length, mitochondrial DNA copy number, and GPX activity in our study (Tables 1, 2 and 3). For the impact of air and water pollution, it is a limitation of our study to lack data on exposure to pollutants. Future studies with larger sample sizes are necessary to investigate the relationship of prenatal thyroid hormones and aging markers at birth while also considering the impact of environmental factors.

## Conclusion

Overall, our study indicated a reverse relationship between cord blood leukocytes telomere length and serum TSH levels, accompanied by increased GPX activity. In addition, we observed lower levels of mitochondrial DNA copy number in association with higher fT4 thyroid hormone. Our results provide data on the effects of the level of prenatal thyroid hormones on age-related factors which will potentially exert life-long influence on the individual’s health. Therefore, our data implicates the importance of tightly regulating the level of TSH and fT4 during pregnancy and suggest the transgenerational effects of thyroid hormones on aging. Future follow-up studies on larger population sizes are required to validate our findings. The underlying molecular mechanism of the relation between thyroid hormones and molecular aging parameters can be better understood by future analyses that provide a comprehensive view of the status of various oxidative stress markers, mitochondrial biogenesis status, telomerase activity, and the level of DNA damage.

## Data Availability

The datasets generated and analyzed during the current study are not publicly available but are available from the corresponding author at a reasonable request.
